# Bleeding risk assessment in patients with new-onset atrial fibrillation after coronary surgery: a proof-of-concept study

**DOI:** 10.1093/ehjcvp/pvag040

**Published:** 2026-06-16

**Authors:** Mary Rezk, Amar Taha, Andreas Martinsson, Susanne J Nielsen, Tomas Gudbjartsson, Lennart Bergfeldt, Florian E M Herrmann, Emma C Hansson, Anders Jeppsson

**Affiliations:** Department of Cardiothoracic Surgery, Sahlgrenska University Hospital, Gothenburg 413 45, Sweden; Department of Molecular and Clinical Medicine, Institute of Medicine, Sahlgrenska Academy, University of Gothenburg, Gothenburg 413 90, Sweden; Department of Molecular and Clinical Medicine, Institute of Medicine, Sahlgrenska Academy, University of Gothenburg, Gothenburg 413 90, Sweden; Department of Cardiology, Sahlgrenska University Hospital, Gothenburg 413 45, Sweden; Department of Molecular and Clinical Medicine, Institute of Medicine, Sahlgrenska Academy, University of Gothenburg, Gothenburg 413 90, Sweden; Department of Cardiology, Sahlgrenska University Hospital, Gothenburg 413 45, Sweden; Department of Cardiothoracic Surgery, Sahlgrenska University Hospital, Gothenburg 413 45, Sweden; Department of Molecular and Clinical Medicine, Institute of Medicine, Sahlgrenska Academy, University of Gothenburg, Gothenburg 413 90, Sweden; Department of Cardiothoracic Surgery, Landspitali University Hospital, Reykjavik 101, Iceland; Department of Molecular and Clinical Medicine, Institute of Medicine, Sahlgrenska Academy, University of Gothenburg, Gothenburg 413 90, Sweden; Department of Cardiology, Sahlgrenska University Hospital, Gothenburg 413 45, Sweden; Department of Molecular and Clinical Medicine, Institute of Medicine, Sahlgrenska Academy, University of Gothenburg, Gothenburg 413 90, Sweden; Department of Cardiac Surgery, LMU University Hospital, LMU Munich, Munich 81377, Germany; DZHK (German Centre for Cardiovascular Research) partner site Munich Heart Alliance, Munich 80802, Germany; Department of Cardiothoracic Surgery, Sahlgrenska University Hospital, Gothenburg 413 45, Sweden; Department of Molecular and Clinical Medicine, Institute of Medicine, Sahlgrenska Academy, University of Gothenburg, Gothenburg 413 90, Sweden; Department of Cardiothoracic Surgery, Sahlgrenska University Hospital, Gothenburg 413 45, Sweden; Department of Molecular and Clinical Medicine, Institute of Medicine, Sahlgrenska Academy, University of Gothenburg, Gothenburg 413 90, Sweden

**Keywords:** Post-operative atrial fibrillation, Bleeding risk, Anticoagulation

## Abstract

**Aims:**

Current guidelines recommend considering long-term oral anticoagulation in patients with new-onset post-operative atrial fibrillation (POAF) after cardiac surgery, balancing stroke and bleeding risk. However, no specific approach to bleeding risk assessment is provided. We explored in a proof-of-concept study whether a bleeding risk score can identify patients with POAF after coronary artery bypass grafting (CABG) with increased risk of post-discharge major bleeding.

**Methods and results:**

This observational cohort study included 4436 patients with POAF after CABG in 2009–2020 without oral anticoagulation. The four-item PRECISE-DAPT score (based on age, creatinine clearance, preoperative haemoglobin concentration, and previous bleeding) was calculated for all patients. Bleeding risk was defined as high (≥25 points), medium (16–24 points), or low (≤15 points). Associations between bleeding risk and major bleeding events during the first post-operative year were assessed by Cox regression. Discrimination was evaluated with C-statistics, and calibration was assessed by comparing expected and observed bleeding rates. Major bleeding occurred in 2.1% of patients during the first year. The score classified 36.0% of patients as high bleeding risk. The hazard ratio for high vs. low bleeding risk was 4.81 (95% CI 2.59–8.96). The area under the receiver operating characteristic curve was 0.68 (95% CI 0.63–0.73). Calibration showed good agreement between expected and observed bleeding events in patients with an annual bleeding risk up to 7%.

**Conclusion:**

A bleeding risk score can be used to stratify patients with POAF after CABG into groups with different post-discharge bleeding risk. Further studies are necessary to identify the optimal risk score and its role in oral anticoagulation decision pathway to improve clinical outcomes.

## Graphical Abstract

**Graphical Abstract:**
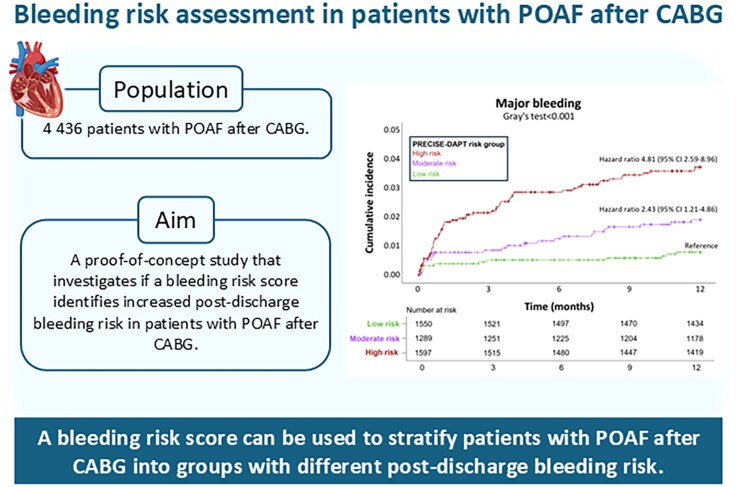

## Introduction

New-onset post-operative atrial fibrillation (POAF) is a common early condition after cardiac surgery, traditionally reported in ∼30% of patients after isolated coronary artery bypass grafting (CABG).^1,2^ Two recent studies with implantable rhythm monitors demonstrate a higher incidence and, importantly, a very low atrial fibrillation (AF) burden after hospital discharge.^3,4^ While POAF usually is transient, previous studies have shown an association between POAF and increased long-term risk for ischaemic stroke.^5,6^ This association raises important questions about its management, particularly regarding long-term oral anticoagulation (OAC) therapy, whose indications remain a subject of debate.^7,8^

Current European Society of Cardiology’s (ESC) AF guidelines state that long-term OAC should be considered in patients with POAF after cardiac surgery if they are at risk for stroke, evaluating the bleeding risk (Class 2A, LoE B).^9^ The European Association for Cardiothoracic Surgery (EACTS) has a class 2B recommendation for patients with POAF in sinus rhythm at discharge and OAC therapy for at least 4 weeks in patients with persistent POAF at discharge, followed by re-evaluation (Class I, LoE B).^10^ The 2023 American College of Cardiology/American Heart Association (ACC/AHA) guidelines state that it is reasonable to administer OAC for 60 days in patients with POAF whose bleeding risk is acceptable, followed by a reassessment of the need for long-term anticoagulation (Class 2A LoE B-NR).^11^ However, none of the guidelines give recommendations on how to assess bleeding risk in patients with POAF.

A potential tool for bleeding risk stratification is a bleeding risk score. Several different scores have been developed and validated for specific patient categories, such as the HAS-BLED score for patients on warfarin^12^ and the PRECISE-DAPT score for patients receiving dual antiplatelet therapy (DAPT) after percutaneous coronary intervention (PCI).^13^ The original PRECISE-DAPT score was developed in patients undergoing PCI treated with DAPT, and its use is endorsed by a Class I recommendation in the 2018 ESC/EACTS guidelines on myocardial revascularization.^14^ In contrast, no validated and widely adopted bleeding-risk stratification tool has been established for use in the CABG or POAF settings. The aim of this proof-of-concept study is therefore to explore whether a bleeding risk score can stratify post-discharge bleeding risk during the first 12 post-operative months in patients with POAF after CABG.

## Methods

### Statements

This study was conducted in accordance with the Declaration of Helsinki and approved by the Swedish Ethical Review Authority (registration number 2021-00122, approved 31 March 2021). The committee waived the need for individual patient consent. The manuscript follows the recommendations in the Strengthening the Reporting of Observational Studies document (STROBE).^15^ Patients or the public were not involved in the design, conduct, reporting, or dissemination plans of this study.

### Study design and patient population

**Figure 1:**
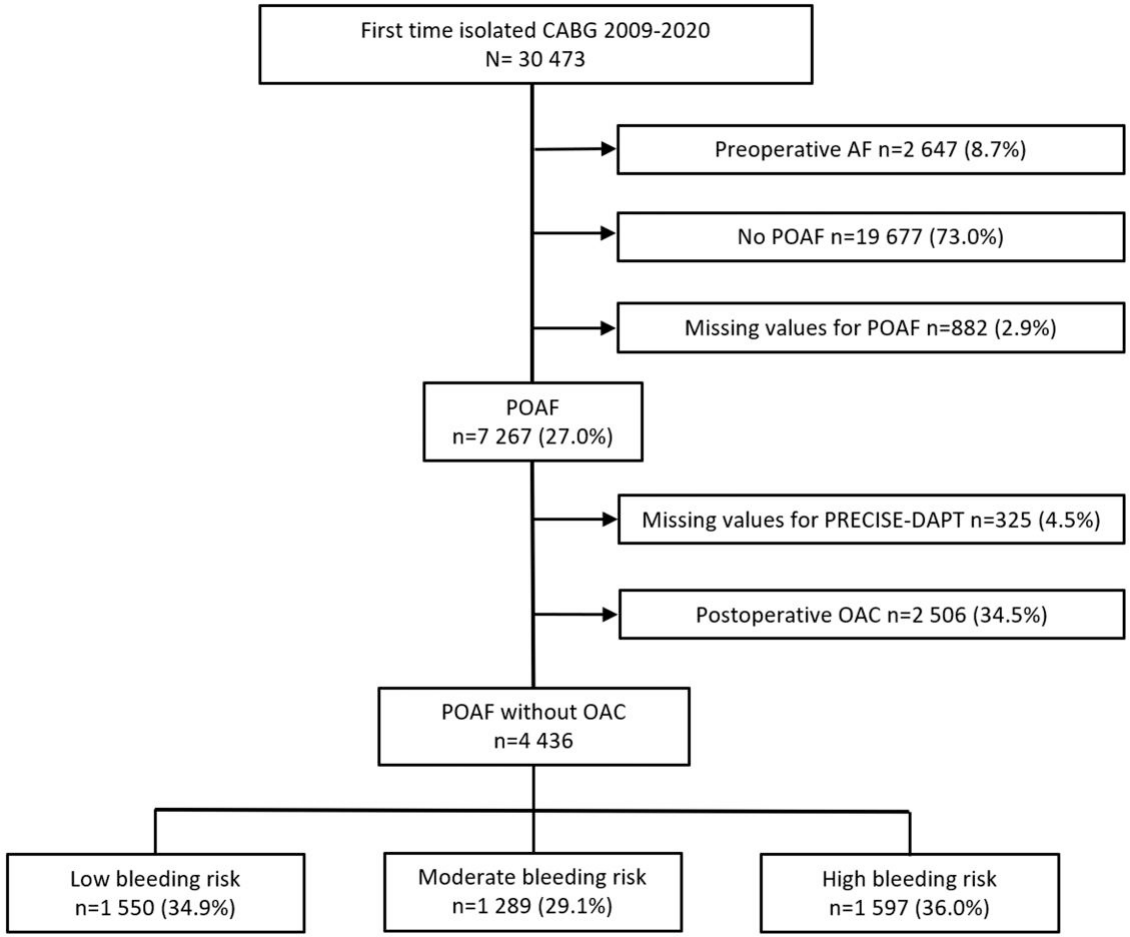
A flow chart of included and excluded patients in the study. AF: Atrial fibrillation; CABG: Coronary artery bypass grafting; OAC: oral anticoagulation; POAF: post-operative atrial fibrillation.

This observational nationwide cohort study includes all patients ≥18 years in Sweden who developed POAF after undergoing first-time isolated CABG from 2009 to 2020. POAF was defined as any new-onset AF during the index hospitalization in a patient with no documentation of AF in national registries prior to CABG. Exclusion criteria were OAC treatment during the first post-operative year after discharge and missing variables for the calculation of the bleeding risk score (PRECISE-DAPT). Patients with post-operative OAC therapy were excluded to mimic a real-life clinical scenario where a clinician with a patient with new-onset POAF must decide whether to initiate OAC treatment or not before discharge. Patients with POAF were divided into bleeding risk groups according to the score. *Figure 1* is a flow chart of included and excluded patients.

The score was calculated based on the patients’ clinical characteristics at baseline, which were prospectively collected in the registries. All patients were followed until death, emigration, or 12 months after discharge, whichever occurred first. The primary outcome was post-discharge major bleeding during the first 12 post-operative months, assessed when associated with hospitalization or death and reported in the National Patient Registry as the primary diagnosis according to ICD-10 codes.

### Data sources

Information on patients was obtained from five nationwide mandatory registries and databases in Sweden. The patients were identified in the Swedish Cardiac Surgery Registry,^16^ a part of the SWEDEHEART registry,^17^ which covers all open cardiac surgery procedures in Sweden since 1992. Preoperative clinical data, preoperative AF, post-operative complications, and information on POAF were obtained from the Swedish Cardiac Surgery Registry and the National Patient Registry. The National Patient Registry was established in 1964 and contains all diagnoses, length of stay, and discharge dates from all hospitalizations and hospital outpatient visits in Sweden.^18^ Information on baseline characteristics and outcome variables was identified using the ICD-10. The ICD codes used are listed in Supplementary material online, *Table S1*.

The Dispensed Drug Registry was used to obtain information on dispensed prescriptions for post-operative oral anticoagulants and antiplatelet agents by Anatomical Therapeutic Chemical (ATC) codes. Supplementary material online, *Table S2* gives the ATC codes used. Data on mortality were collected from the Swedish Cause of Death Register and emigration status from the Swedish Population Register. Individual patient data from all registries were combined into a single database using the unique personal identification numbers assigned to every resident in Sweden.

### PRECISE-DAPT

The four-item PRECISE-DAPT score was originally designed to predict out-of-hospital bleeding risk in patients treated with DAPT after PCI. The score incorporates information about age, preoperative haemoglobin concentration, estimated creatinine clearance, and history of major bleeding^19^ and has been externally validated.^20^ In this study, creatinine clearance and haemoglobin concentration were based on samples collected at admission for CABG surgery. History of major bleeding was collected from the National Patient Registry and defined as hospitalization due to bleeding any time before admission. A recent study in an unselected cohort of CABG patients on single or DAPT showed that the four-item score has comparable accuracy in patients undergoing CABG as in patients undergoing PCI.^21^ Patients with POAF were classified into three risk categories according to the original four-item score: Very low and low bleeding risk (score 0–15), moderate bleeding risk (score 16–24), and high bleeding risk (score ≥ 25).^19^

### Statistics

Continuous variables are expressed as means with standard deviations and compared using independent samples *t*-test. Categorical values are expressed as numbers with percentages and compared using chi-squared tests. Cumulative incidence of major bleeding was calculated using the Aalen–Johansen estimator, which accounts for the competing risk of death. Grey’s test was used to assess differences across score strata. Hazard ratios (HRs) and a 95% confidence interval (CI) were calculated using Cox proportional hazard analysis to determine the association between endpoint and risk categories, using low-risk patients as the reference group. A sensitivity analysis using the Fine-Grey method, which accounts for the competing risk of death, was also applied for the comparison between the bleeding risk groups. Analyses were also performed separately for patients treated with no antiplatelet therapy, mono-antiplatelet therapy, and DAPT. The proportional hazards assumption was assessed using Shoenfeld residuals and log-minus-log survival plots. C-statistics from receiver operating characteristic (ROC) curves were utilized to evaluate the score’s prognostic accuracy for 1-year bleeding. The area under the ROC curve is presented with 95% CI, where the area <0.60 indicates poor discrimination, 0.60–0.75 suggests possibly helpful discrimination, and >0.75 signifies clearly useful discrimination.^22^ Model calibration was assessed using a calibration plot, which compares predicted probabilities of major bleeding with observed events. A bias-corrected calibration curve was implemented by bootstrapping. Calibration was evaluated using the calibration slope and intercept, where a slope of 1 and an intercept of 0 indicate perfect calibration.^23^ The estimated probability of major bleeding was determined based on the original PRECISE-DAPT score. The clinical utility of the score was evaluated using decision curve analysis (DCA), which quantifies the net benefit across a range of threshold probabilities.^24^ DCA accounts for both sensitivity and specificity while incorporating the relative harm of true and false positives.

A *P*-value of <0.05 was considered statistically significant. Data were processed using IBM SPSS Statistics for Windows, version 29.0 (IBM Corp., Armonk, NY, USA) and R version 4.5.0 (R Foundation for Statistical Computing, Vienna, Austria).

## Results

### Study patients

The study included a total of 30 473 patients. After exclusion of patients with preoperative AF, patients with missing information about POAF and patients without POAF, 7267 patients with POAF remained. A total of 2506 (34.5%) patients were excluded due to OAC during the first year and 325 (4.5%) due to missing values for calculation of the PRECISE-DAPT score, leaving 4436 patients with POAF and without early initiated OAC included in the final analysis (*Figure 1*). Supplementary material online, *Table S3* reports baseline characteristics of included and excluded patients. Patients with OAC were older and had a higher incidence of heart failure and a lower incidence of previous major bleeding. PRECISE-DAPT and CHA_2_DS_2_-VASc score were similar in patients with and without early initiated OAC.

*Table 1* presents preoperative patient characteristics according to bleeding risk. Compared with patients with low bleeding risk, those with high bleeding risk tended to be older, had lower preoperative haemoglobin and lower creatinine clearance or renal failure, and a higher frequency of diabetes, heart failure, peripheral vascular disease, previous stroke, previous major bleeding, and myocardial infarction. The use of DAPT at discharge was more common in patients with low bleeding risk (see Supplementary material online, *Table S4*). Overall, 64/4436 patients (1.4%) died during the first 12 post-operative months after discharge.

**Table 1:** Preoperative baseline characteristics of patients with POAF according to bleeding risk

Variables	All patients *n* = 4436	Bleeding risk		
		High risk *n* = 1597	Moderate risk *n* = 1289	Low risk *n* = 1550
Age, years	69.3 ± 7.8	72.7 ± 7.3	72.1 ± 5.4	63.4 ± 6.6
Female	788 (17.8)	401 (25.1)	222 (17.2)	165 (10.6)
Body mass index, kg/m^2^	27.7 ± 6.3	27.6 ± 6.2	27.4 ± 8.0	28.2 ± 4.3
Indication for surgery				
Acute coronary syndrome	2869 (64.7)	1101 (68.9)	829 (64.3)	939 (60.6)
Chronic coronary disease	1567 (35.3)	496 (31.1)	460 (35.7)	611 (39.4)
Preoperative haemoglobin, g/L	138 ± 15	131 ± 17	139 ± 13	143 ± 11
Creatinine clearance, mL/min	85 ± 30	69 ± 28	78 ± 16	109 ± 26
Diabetes	1290 (29.1)	546 (34.2)	312 (24.2)	432 (27.9)
Heart failure	536 (12.1)	267 (16.7)	133 (10.3)	136 (8.8)
Peripheral vascular disease	400 (9.0)	213 (13.3)	107 (8.3)	80 (5.2)
Previous stroke	316 (7.1)	185 (11.6)	80 (6.2)	51 (3.3)
Previous major bleeding^a^	288 (6.5)	288 (18.0)	0	0
Previous myocardial infarction	2188 (49.3)	891 (55.8)	597 (46.3)	700 (45.2)
Previous renal failure	185 (4.2)	160 (10.0)	14 (1.1)	11 (0.7)
Previous percutaneous coronary intervention	920 (20.7)	343 (21.5)	245 (19.0)	332 (21.4)
PRECISE-DAPT score				
Mean	24 ± 17	43 ± 14	20 ± 3	10 ± 4
Median	19 (13–32)	42 (30–52)	19 (17–22)	11 (7–13)
CHA_2_DS_2_-VASc score				
Mean	4.1 ± 1.5	4.7 ± 1.5	4.1 ± 1.4	3.5 ± 1.2
Median	4 (3–5)	5 (4–6)	4 (3–5)	4 (2–4)

Mean ± standard deviation or numbers (%). ^a^Bleeding that required hospitalization.

### PRECISE-DAPT and bleeding risk

In total, 94/4436 (2.1%) patients experienced at least one major bleeding event during the first post-operative year after discharge. The overall incidence of major bleeding adjusted for the competing risk of death is depicted in Supplementary material online, *Figure S1*.

**Figure 2:**
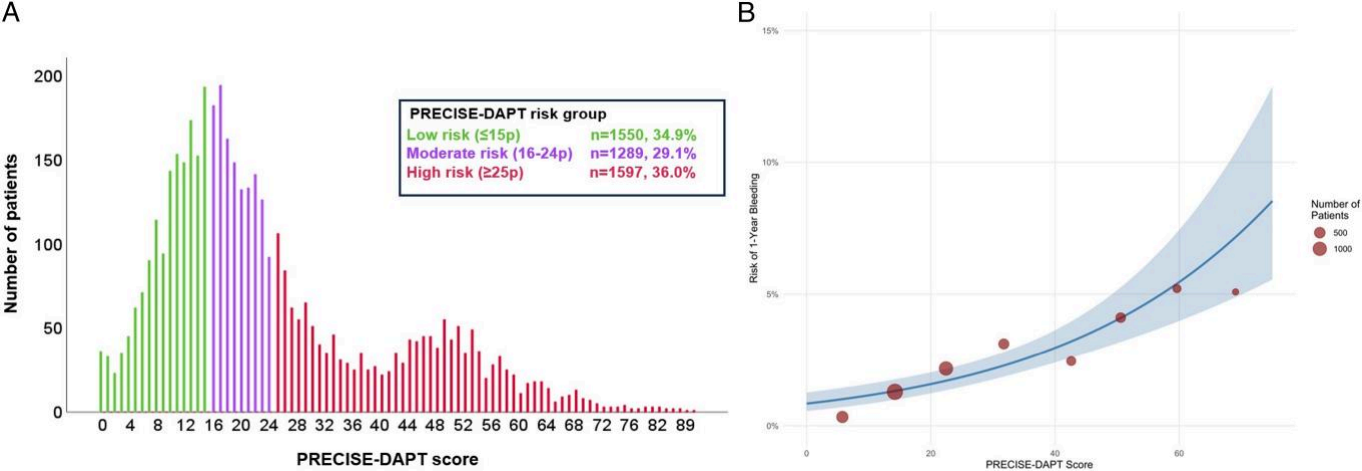
(*A*) The distribution of the PRECISE-DAPT risk score in patients with POAF after CABG. (*B*) Observed 1 year bleeding risk stratified by PRECISE-DAPT.

**Figure 3:**
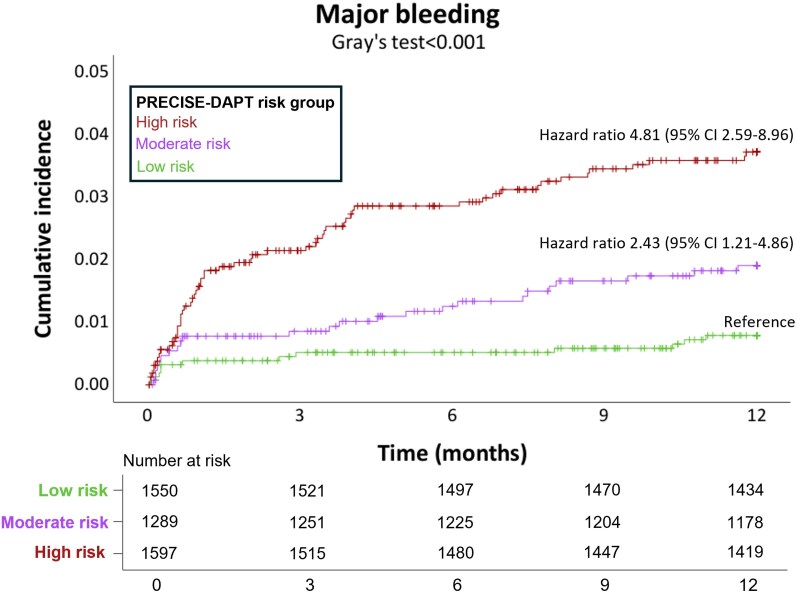
The cumulative incidence of major bleeding in relation to PRECISE-DAPT risk group. Low-risk patients used as reference. CI: confidence interval.

The mean bleeding risk score was 24 ± 17 points, and the median score was 19 (interquartile range: 13–32). The distribution of the risk score is shown in *Figure 2A*. *Figure 2B* illustrates the observed bleeding risk during the first post-operative year stratified by the score. A total of 1597/4436 (36.0%) patients were estimated to be at high risk for major bleeding after discharge. Major bleeding occurred in 0.8% of patients with low risk, 1.9% with moderate risk, and 3.6% with high risk (see Supplementary material online, *Table S5*). *Figure 3* shows the cumulative incidences of major bleeding by risk group. The HRs were 4.81 (95% CI 2.59–8.96, *P* < 0.001) for the group with high vs. low risk and 2.43 (95% CI 1.21–4.86, *P* = 0.012) for moderate vs. low risk, respectively. The sensitivity analysis taking the competing risk of death into account yielded almost identical results as the Cox regression model (sub-distribution HR: 4.77 (2.56–8.88 for high vs. low risk and 2.42 (1.21–4.84) for moderate vs .low risk). The Cox regression HRs were consistent in patients treated with no antiplatelet therapy, mono-antiplatelet therapy, and DAPT, as depicted in Supplementary material online, *Figure S2*.

**Figure 4:**
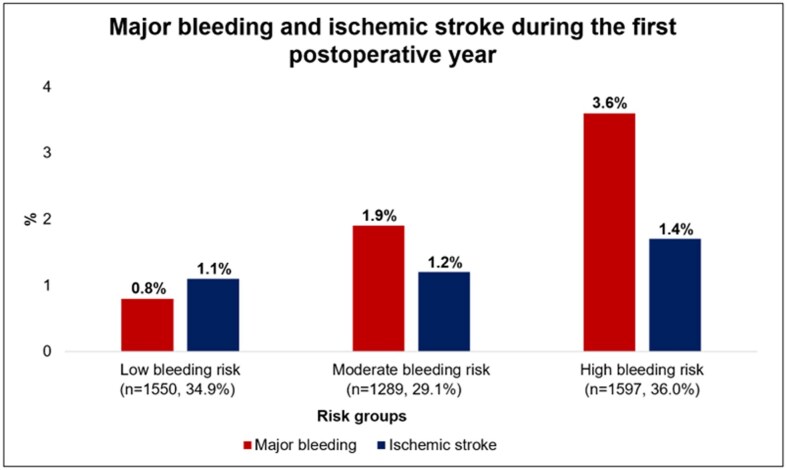
The incidence of major bleeding and ischemic stroke during the first post-operative year in different risk groups.

The incidence of post-discharge ischaemic stroke during the first post-operative year was 1.1%, 1.2%, and 1.4% in the low, moderate, and high bleeding risk groups, respectively (*Figure 4*).

### Discrimination accuracy, calibration, and clinical usefulness

**Figure 5:**
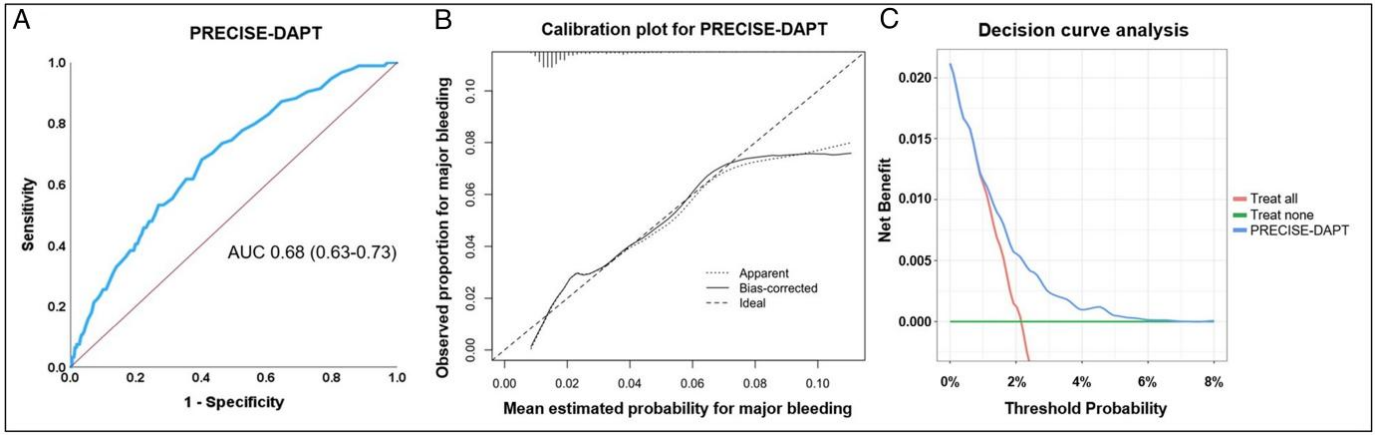
Prediction models (*A*) The area under the ROC curve. (*B*) Calibration plot with bootstrapping (dotted line). (*C*) Decision curve analysis for PRECISE-DAPT where green line is treat none, red line is treat all, and blue line is the PRECISE-DAPT score. AUC: area under the curve.

The area under the ROC curve for the score was 0.68 (0.63–0.73) (*Figure 5A*). The calibration plot (*Figure 5B*) showed good agreement between predicted and observed probabilities, with an intercept of 0.074 and a slope of 1.018. However, the score overestimated bleeding risk in patients with a predicted risk >7%.

The DCA for the score shows that the net benefit of the score was higher than both the ‘treat all’ and ‘treat none’ for threshold probabilities between ∼1% to 4.5% (*Figure 5C*). Beyond this range, the net benefit of the score declines and approaches that of the ‘treat none’.

## Discussion

The key finding of this large, nationwide, observational cohort study was that the bleeding risk score identified patients with POAF after CABG with an increased bleeding risk with moderate accuracy and satisfactory calibration, comparable to the accuracy previously reported in patients undergoing PCI or CABG. The study demonstrates that a bleeding risk score can identify post-discharge bleeding risk in POAF patients after CABG, but it does not prove that using this score to guide anticoagulation improves clinical outcomes.

Current ESC AF guidelines state that OAC therapy should be considered in patients with POAF after cardiac surgery if they are at risk for stroke (Class IIa, LoE B), balancing bleeding risk.^9^ The current ACC/AHA guidelines recommend OAC for 60 days post-operatively in patients with POAF after cardiac surgery, provided it is safe regarding bleeding risk.^11^ They also recommend reevaluating the need for long-term OAC therapy after 60 days of treatment (Class IIa, LoE B-NR). The 2024 EACTS perioperative medication guidelines have a stronger recommendation for OAC in patients with persistent AF at discharge (Class 1, LoE B) and a weaker recommendation for patients in sinus rhythm at discharge (Class 2B, LoE B)^10^ and recommend re-evaluation of the indication for OAC after 4 weeks. Importantly, OAC decisions in patients with POAF are also based on the clinical presentation of POAF, such as duration, persistence at discharge, and recurrence during follow-up. However, the definition of POAF varies between studies. A more universal definition of POAF and a clearer risk stratification are warranted to guide OAC decisions. The uncertainty of guideline recommendations is explained by the lack of randomized controlled trials specifically addressing patients with POAF. Although there are observational studies indicating that OAC therapy in patients with POAF is associated with reduced risk for thromboembolic events,^2^ several other studies, including a large meta-analysis, have reported that OAC treatment in patients with POAF is associated with an increased risk of major bleeding,^1,25–27^ without a reduction in thromboembolic risk. These results can be explained by the very low AF burden after discharge in POAF patients, recently demonstrated in two prospective studies using implantable heart rhythm monitors.^3,4^

In the present study, the bleeding risk score identified approximately one-third of patients with POAF after CABG to have a high risk of bleeding. These patients had an almost fivefold relative increase in the risk of major bleeding compared with patients at low bleeding. If these patients have sinus rhythm at discharge and a normal stroke risk (i.e. CHA_2_DS_2_-VASc score of 3–4), it appears reasonable to shorten the period of OAC or withhold it unless AF recurs. The proportion of patients at high risk for major bleeding (36.0%) was slightly higher than that previously reported in patients undergoing PCI,^28^ but comparable to CABG patients on DAPT (32.9%) or antiplatelet monotherapy (38.2%).^21^ In this study, the score demonstrated a moderate but clinically useful discrimination for major bleeding at 1 year (AUC 0.68), aligning with observations from the IPD-MA derivation and validation studies of PRECISE-DAPT in PCI patients (AUC 0.62–0.73)^28^ and previous CABG studies (AUC 0.62–0.71),^21^ strengthening the external face validity of the current findings. However, the moderate discrimination accuracy suggests a need for improvement and calls for further studies comparing different bleeding risk assessment tools.

The calibration plot demonstrated overall good agreement between predicted and observed major bleeding events, although the risk score overestimated the bleeding risk in patients with very high bleeding risk (>7%). This is important as these are the patients for whom a reliable risk estimate is most needed before prescribing OAC and may lead to underuse of OAC in patients who might still benefit. Therefore, these estimates should be interpreted with caution and in conjunction with the patient individual judgment. The DCA indicates that use of the score provides a positive net benefit compared with a ‘treat all’ or ‘treat none’ approach within a narrow range of threshold probabilities (1–4.5%). The limited benefit is due to the low prevalence of major bleeding events.

The present study has both strengths and weaknesses. Strengths include the large, nationwide study population, and real-life data from validated registries. No patients were lost to follow-up. The limitations include the observational study design and the number of patients excluded due to missing values for bleeding risk score calculation (4.5%). Furthermore, bleeding definitions commonly used in clinical trials, such as the Bleeding Academic Research Consortium bleeding classification or the Thrombolysis in Myocardial Infarction bleeding criteria, could not be used since the registries lack data on transfusions and haemoglobin levels. Instead, hospitalization for bleeding or death due to bleeding was used as in other prospective trials^28^ and observational studies.^1,29,30^ This likely undercounts clinically relevant but non-hospitalized bleeds and makes the absolute observed 1-year risk (2.1% overall, 3.6% in ‘high risk’) look modest. Nevertheless, the low overall incidence of major bleeding and the use of a registry-based bleeding definition may restrict a score’s clinical utility.

We chose to include only patients without OAC in the present analyses. As stated above, this was done to mimic a situation where the clinician is considering OAC before discharge. However, the exclusion of patients on OAC results in that the findings mainly apply to POAF patients whom clinicians judge safe to discharge without OAC. Hence, the results should not be automatically generalized to a wider POAF population, particularly those in whom anticoagulation is strongly considered. On the other hand, mean CHA_2_DS_2_-VASc [Congestive heart failure, Hypertension, Age ≥ 75 years (2 points), Diabetes mellitus, Stroke/transient ischemic attack/thromboembolism (2 points), Vascular disease, Age 65–74 years, Sex category (female)] and PRECISE-DAPT scores did not differ markedly between patients with and without early initiated OAC, indicating that perceived stroke and bleeding risks were not major determinants of OAC initiation.

There are several other alternative bleeding risk scores that could have been used. For this proof-of-concept study, we chose PRECISE-DAPT since it contains the included variables in bleeding risk scores (age, renal function, previous major bleeding, haemoglobin levels), has been validated in CABG patients, and includes data that are available in the national registries we utilized. Other bleeding risk scores, such as HAS-BLED, contain variables that are rarely collected and validated in nationwide registries, such as alcohol consumption and time in therapeutic INR interval for warfarin.

In conclusion, our proof-of-concept study shows that it is possible to use a bleeding risk score to assess post-discharge bleeding risk in patients with POAF after CABG. The utilized score separated patients into groups with different observed risk scores with moderate accuracy and satisfactory calibration. However, the role of risk assessment tools in guiding OAC therapy and improving clinical outcomes in patients with POAF remains to be established. Further studies, preferably prospective, are needed to examine the role of bleeding risk assessment tools and to compare different bleeding risk scores in patients with POAF with or without OAC.

## Supplementary Material

pvag040_Supplementary_Data

## Data Availability

The data in this study will be provided upon reasonable request with approval from the concerned authorities.
