# Supplementary material for: Bleeding risk assessment in patients with new-onset atrial fibrillation after coronary surgery: a proof-of-concept study
Source: Eur Heart J Cardiovasc Pharmacother. 2026 Jun 16;12(5):356–64. doi: 10.1093/ehjcvp/pvag040 (PMC13588634; doi:10.1093/ehjcvp/pvag040)
Supplement: Supplementary file 1 [file pvag040_supplementary_data.docx]

Supplementary Material to

**Bleeding risk assessment in patients with new-onset atrial fibrillation after coronary surgery: A proof-of-concept study**

Mary Rezk, Amar Taha, Andreas Martinsson, Susanne J. Nielsen, Tomas Gudbjartsson, Lennart Bergfeldt, Florian EM Herrman, Emma C. Hansson, Anders Jeppsson

**Content**

Table S1	ICD-10 codes for diagnoses used in the study.

Table S2	ATC-codes used in the study

Table S3	Preoperative baseline characteristics of included and excluded
 patients

Table S4	Antiplatelet therapy in relation to bleeding risk and stroke risk

Table S5	Number of events, event rate per 100 patient years and hazard ratios for the different bleeding risk groups

Figure S1	The overall incidence of major bleeding adjusted for the competing risk of death

Figure S2	The cumulative incidence of major bleeding in relation to PRECISE-DAPT risk group in patients treated with mono or dual antiplatelet therapy

**SUPPLEMENTARY TABLES**

**Supplementary Table S1.** ICD-10 codes for diagnoses used in the study.

|  | **ICD-10** |
| --- | --- |
| **Diabetes** | E10-E14 |
| **Heart failure** | I42, I43, I50 |
| **Hypertension** | I10-15 |
| **Peripheral vascular disease** | I70-I74, I77 |
| **Myocardial infarction** | I21, I22 |
| **PCI** | FNG |
| **Atrial fibrillation** | I48 |
| **Stroke** | I61-I64, I69 |
| **Transitory ischemic attack** | G45, I66, I65 |
| **Renal disease** | N17-19, DR016, DR024, KAS10, KAS20 |
| **Bleeding** | D50, D62, D68-D69, H356, H922,  I230, I312, I60-I62, I690-I692,I850, I983, J942, K221, K226, K25-K28, K290, K625, K661, K920, K921, K922, M250, N02, N398, N421, N501A, N939, N950, R31, R040-R042, R048, R049 |

**Supplementary Table S2.** ATC-codes used in the study.

|  | **ATC-codes** |
| --- | --- |
| **Oral anticoagulants** | B01AA03, B01AE07, B01AF01, B01AF02, B01AF03 |
| **Acetylsalicylic acid** | B01AC06 |
| **Clopidogrel** | B01AC04 |
| **Prasugrel** | B01AC22 |
| **Ticagrelor** | B01AC24 |

**Supplementary Table S3.** Preoperative baseline characteristics of included and excluded patients.

| **Variables** | **All patients**  **N=4336** | **POAF patients**  **with OAC**  **n=2506** | **Missing**  **PRECISE-DAPT**  **n=325** |
| --- | --- | --- | --- |
| Age, years | 69.3±7.8 | 72.7±7.3 | 69.7±8.5 |
| Female | 788 (17.8) | 457 (18.2) | 57 (17.5) |
| Body Mass Index, kg/m^2^ | 27.7±6.3 | 27.8±5.4 | 27.2±4.1 |
| Indication for surgery  Acute coronary syndrome  Chronic coronary disease | 2869 (64.7)  1567 (35.3) | 1623 (64.8)  883 (35.2) | 231 (71.1)  94 (28.9) |
| Preoperative hemoglobin, g/L | 138±15 | 137±15 | 128±21 |
| Creatinine clearance, mL/min | 85±30 | 84±29 | 85±30 |
| Diabetes | 1290 (29.1) | 715 (28.5) | 92 (28.3) |
| Heart Failure | 536 (12.1) | 372 (14.8) | 42 (12.9) |
| Peripheral vascular disease | 400 (9.0) | 256 (10.2) | 31 (9.5) |
| Previous stroke | 316 (7.1) | 177 (7.1) | 21 (6.5) |
| Previous major bleeding* | 288 (6.5) | 132 (5.3) | 14 (4.3) |
| Previous myocardial Infarction | 2188 (49.3) | 1256 (50.1) | 172 (52.9) |
| Previous renal failure | 185 (4.2) | 100 (4.0) | 9 (2.8) |
| Previous percutaneous coronary intervention | 920 (20.7) | 529 (21.1) | 56 (17.2) |
| PRECISE-DAPT score  Mean  Median | 24±17  19 (13-32) | 26±16  21 (15-33) | - |
| CHA_2_DS_2_-VASc score  Mean  Median | 4.1±1.5  4 (3-5) | 4.3±1.5  3 (3-4) | 3.5±1.4  3 (2-4) |
| *Mean ± standard deviation or numbers (%). *Bleeding that required hospitalization.* | | | |

**Supplementary Table S4.** Antiplatelet therapy at discharge in relation to bleeding risk.

|  | **Monotherapy** | **p-value** | **DAPT** | **p-value** |
| --- | --- | --- | --- | --- |
| **Bleeding risk group** |  | 0.16 |  | 0.004 |
| **Low** | 1012/1550 (65.3%) |  | 437/1550 (28.2%) |  |
| **Medium** | 886/1289 (68.8%) |  | 322/1289 (25.0%) |  |
| **High** | 1083/1597 (67.8%) |  | 369/1597 (23.1%) |  |
|  |  |  |  |  |
| *DAPT: Dual antiplatelet therapy* | | | | |

**Supplementary Table S5.** Number of events, event rate per 100 patient years and hazard ratios for the different PRECISE-DAPT risk groups.

| **PRECISE-DAPT risk group** | **Number of patients (%)** | **Number of events (%)** | **Event rate per 100 PY** | **Hazard ratio (95% CI)**  **p-value** |
| --- | --- | --- | --- | --- |
| **Low bleeding risk**  **(≤15 points)** | 1550 (34.9) | 12 (0.8) | 0.8 | 1(reference) |
| **Moderate bleeding risk**  **(16-24 points)** | 1289 (29.1) | 24 (1.9) | 2.0 | 2.43 (1.21-4.86) 0.012 |
| **High bleeding risk**  **(≥25 points)** | 1597 (36.0) | 58 (3.6) | 3.9 | 4.81 (2.59–8.96)  <0.001 |
| *Incidence rate per 100 person-years, and Hazard ratios with 95% CI. Low risk is used as reference. CI: Confidence interval; PY: Patient years* | | | | |

**SUPPLEMENTARY FIGURES**

**Supplementary Figure S1.** The overall cumulative incidence of major bleeding adjusted for the competing risk of death.

**Supplementary Figure S2.** The cumulative incidence of major bleeding in relation to PRECISE-DAPT risk group in patients treated with mono-antiplatelet therapy (Panel A), dual anti-platelet therapy (Panel B) or no antiplatelet therapy (Panel C). Low-risk patients used as reference. CI: Confidence interval.
